# Comparison of RNAi Sequences in Insect-Resistant Plants to Expressed Sequences of a Beneficial Lady Beetle: A Closer Look at Off-Target Considerations

**DOI:** 10.3390/insects8010027

**Published:** 2017-03-01

**Authors:** Margaret L. Allen

**Affiliations:** Biological Control of Pests Research Unit, Agricultural Research Service, United States Department of Agriculture, Stoneville, MS 38776, USA; meg.allen@ars.usda.gov; Tel.: +1-662-686-3647

**Keywords:** risk assessment, beneficial organism, genetic pest control, lady beetle

## Abstract

Sequences obtained from transcriptomes of the lady beetle *Coleomegilla maculata* were compared to those designed for incorporation into crops. Searches of the transcriptomes identified sequences as the most likely to be closely similar to the sequences described in RNAi plant incorporated products. Some proposed prime RNAi pest management targets were also used to identify predicted orthologs from *C. maculata*. The lady beetle sequences were aligned with sequences from corn rootworms and Colorado potato beetles and, as appropriate in the case of targets, regions of similarity were compared with the genetic model organism for beetles, *Tribolium castaneum*. Some high levels of nucleotide identity were identified, particularly with an actin-derived sequence from Colorado potato beetle. This actin-derived sequence shared identical sequences with the lady beetle and a parasitic wasp.

## 1. Introduction

One of the most promising emerging insect pest control technologies is based on molecular genetics, and is called RNA interference (RNAi). RNAi is a molecular mechanism that disrupts genes in a target insect prior to the construction of a critical protein, resulting in death of the insect. Double stranded RNA (dsRNA) designed to induce gene knock-down by RNAi in pest insects has been successfully demonstrated and is being developed for implementation in crop protection strategies [1]. RNAi was demonstrated targeting key beetle pests of maize, *Zea mays* L. (corn), the corn rootworms, and a devastating pest of potatoes, *Solanum tuberosum* L. the Colorado potato beetle (CBP), *Leptinotarsa decemlineata* Say (Coleoptera: Chrysomelidae) [2]. Specifically, for field implementation, a sequence from a gene in the genome of the western corn rootworm, *Diabrotica virgifera virgifera* LeConte (Coleoptera: Chrysomelidae) was incorporated into genetically modified maize to combat the damage incurred by the larvae of this beetle and its close relatives to the roots of maize [3,4,5,6]. Another insect targeted for practical RNAi strategies was the Colorado potato beetle (CBP) [7,8]. The potential of RNAi as a tool for pest control is enormous because there are numerous pest targets with many critical genes that could be used in a crop protection, or other pest control, context. The critical genes of the target pest are very likely to be unique to the target because of the degenerate nature of the DNA coding sequence and the variation of genes between organisms. Therefore, a RNAi pest control strategy can be designed that is toxic only to the target insect.

On the other hand, some genes that are crucial to life are highly conserved, or very similar to one another. Genes that are vital to cellular structure and organization, often called “housekeeping genes” may be highly conserved between life forms. This conservation of genes has provided immense benefit to the field of genetics and medicine, because it allows scientists to study genetic mechanisms in one organism, a model organism such as a mouse, a fly, a nematode, or a yeast, and predict mechanisms in humans or other non-model life forms. But if a gene has sufficient similarity at the nucleotide (nt) level, there exists the possibility of cross-species or non-target toxicity or other detrimental effect when RNAi is implemented for pest control. And while the quantity and availability of genomic sequencing data are increasing exponentially, beneficial organisms are infrequently sequenced. Thus, gene sequence-level comparisons for the purpose of predicting off-target effects are mostly unavailable.

The lady beetle *Coleomegilla maculata* De Geer (Coleoptera: Coccinellidae) is a beneficial predator that feeds on the eggs of Colorado potato beetles [9] and also feeds on the pollen of maize [10] and is therefore specifically likely to come into direct contact with the RNAi applications associated with potatoes and maize. A transcriptome analysis of *C. maculata* was prepared in order to identify differences in gene expression based on adult utilization of foods: diets of pollen compared with insect eggs [11]. A primary rationale for selection of a highly inbred population and performing a sequence analysis on this insect was to establish a genetic foundation for further sequencing, with the aim of contributing to a fully sequenced genome of a representative non-target organism. The pair of transcriptomes were not annotated, and therefore could not be easily utilized for non-target analyses in silico when the RNAi products for maize and potato were in development.

This work describes direct comparison of some sequences obtained from the *C. maculata* transcriptomes to those designed for incorporation into crops [12]. Searches of the transcriptomes identified sequences as the most likely to be orthologous to the sequences described in RNAi plant incorporated products. Additionally, eleven proposed prime RNAi pest management targets [13] were used to search for similar sequences in the transcriptomes. The identified *C. maculata* sequences were aligned with sequences from corn rootworms and CPB as appropriate, and in the case of targets, regions of similarity were compared with the genetic model organism for beetles, *Tribolium castaneum* Herbst (Coleoptera: Tenebrionidae).

## 2. Materials and Methods

### Identification of C. maculata Sequences and Comparisons

Transcriptomes of two individual adult insects that were fed two different diets as adults were sequenced and assembled into contigs between 201 base pairs (bp) and >26,000 bp in length [11]. These assembled contigs were compared to National Center for Biological Information (NCBI) Reference RNA sequences (refseq_RNA) [14] and transcriptome shotgun assemblies (TSA) in GenBank using translated BLAST (tBLASTx) algorithm [15]. The spreadsheet generated was sorted and searched to identify sequences that were potentially homologous or orthologous to those evaluated for RNAi pest control sequences targeting *D. v. virgifera* [12] and *L. decemlineata* [7], insects in the same order as *C. maculata*, Coleoptera, and likely to be present in the same North American agroecosystems, maize and potato. Similarly, sequences designated as prime RNAi targets [13] were compared with the paired *C. maculata* transcriptomes after matching the *T. castaneum* sequences to GenBank reference mRNA/cDNA sequences. Candidate *C. maculata* sequences from each assembly were aligned to one another to verify their identity using the NCBI BLAST comparison of two nucleotides setting. Identical or nearly identical sequences were consolidated and reverse transcribed using online sequence manipulation suite (SMS) [16]. Sequences were then loaded into DNAStar EditSeq and aligned using DNAStar MegAlign (Version 12.0. DNASTAR, Madison, WI, USA). The identical portions of the sequences were saved and checked for protein coding translations using the ExPASy Swedish Institute of Bioinformatics (SIB) bioinformatics resource portal translate tool [17]. The translated sequences were compared to characterized genes using the portal’s protein BLAST tool.

## 3. Results

**Figure 1:**
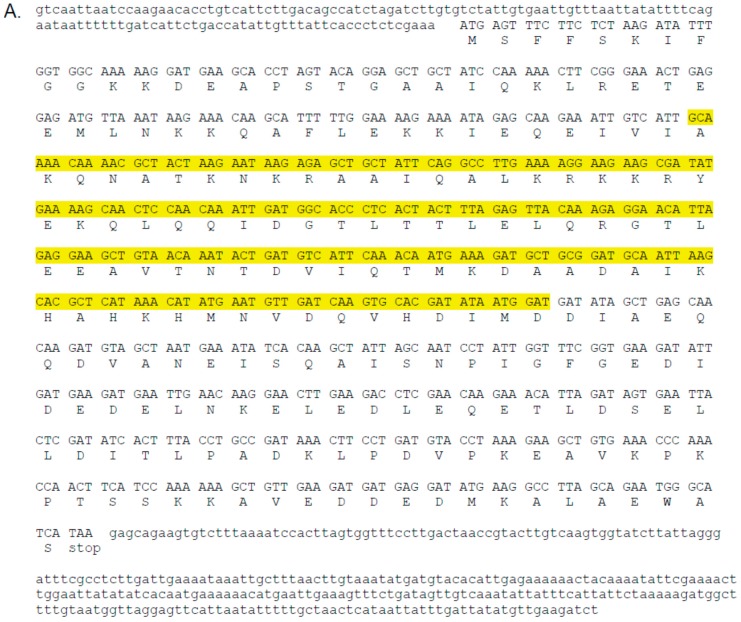

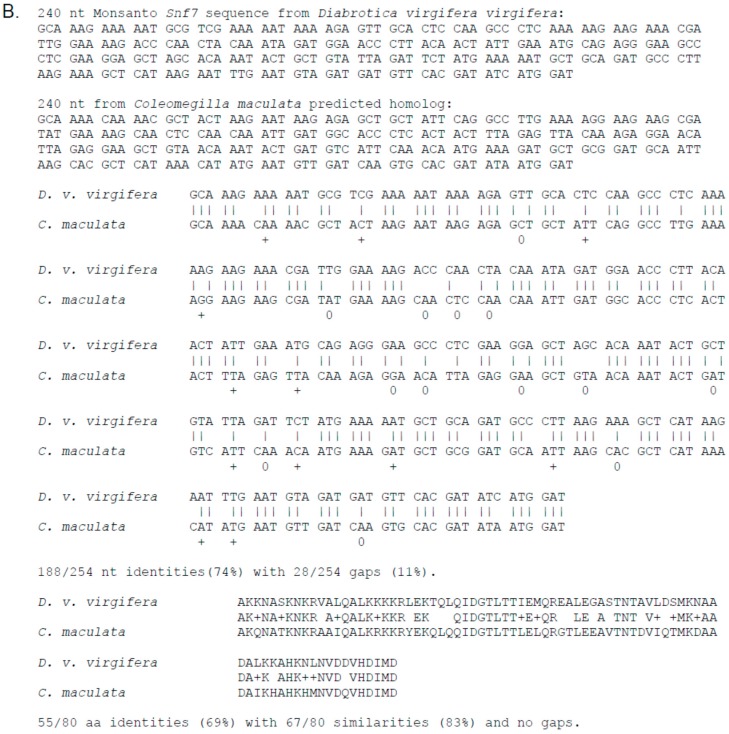
Predicted sequence and translation from *Coleomegilla maculata* transcriptomes most similar to *Diabrotica virgifera virgifera Snf7* used to design rootworm resistant maize. (**A**) Complete cDNA sequence. Highlighted section is the portion of the sequence matching the maize RNAi transgene. Upper case indicates translated sequence, lower case untranslated; (**B**) Alignment of the RNAi transgene regions. Lines between nucleotides indicate identity, identical letters between amino acid sequences indicate identity. The symbol (+) indicates an amino acid substitution by a similar residue, while a (0) or a blank between nucleotide or amino acid letter, respectively, indicates a non-similar substitution. Bottom: amino acid alignment.

A single *C. maculata* sequence with very high similarity to the translated *D. v. virgifera* sequence encoding *Snf7* was found in each of the two transcriptomes. The two putative *C. maculata Snf7* sequences (CmacSnf7) were identical for 1108 nt and encoded a predicted full length protein of 219 amino acids (aa). The translated sequence is shown in Figure 1A. The predicted CmacSnf7 was aligned to the 240 nt used to develop insect resistant transgenic maize. The sequences and alignments of both nt and aa sequences are shown in Figure 1B. The aa identity was 55/80 or 69% and the nt identity was 188/254 or 74%. The nt alignment did not result in any continuous nt identities of >17 nt, the expected length for predicting possible off-target effects [13,18].

**Figure 2:**
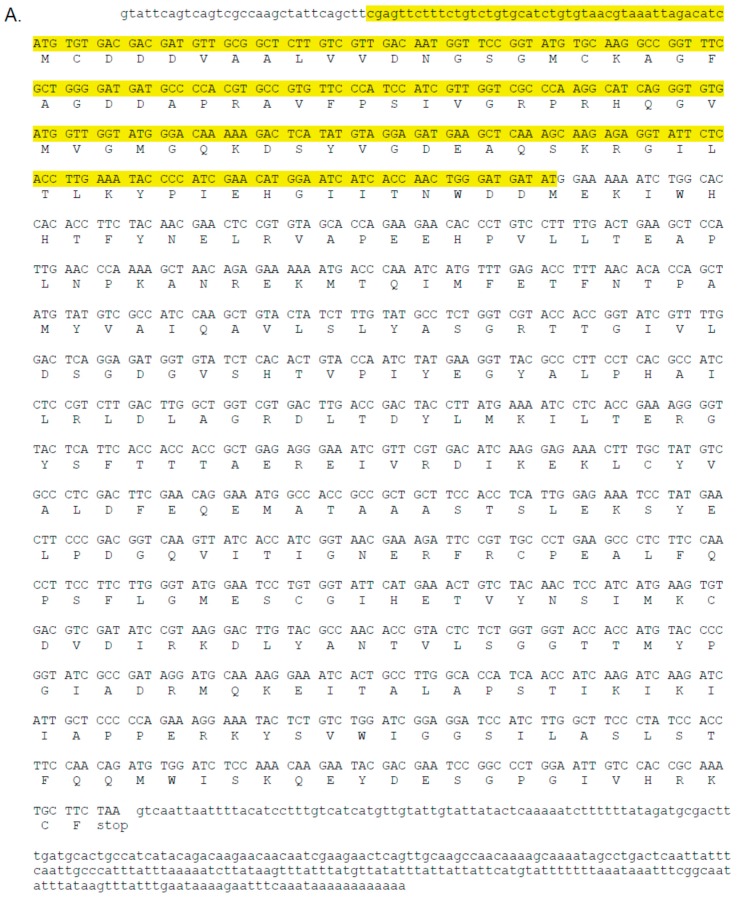

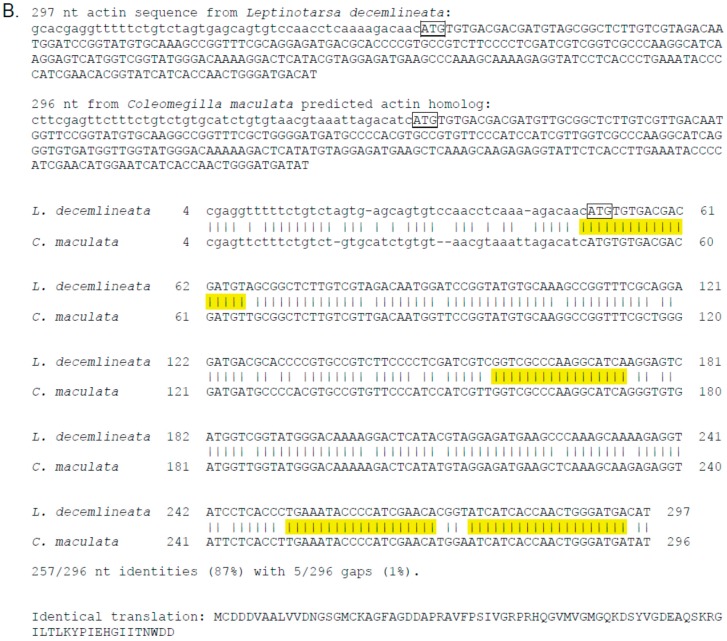
Predicted sequence and translation from *Coleomegilla maculata* transcriptomes most similar to *Leptinotarsa decemlineata* β-actin used to design beetle resistant potato. (**A**) Complete cDNA sequence. Highlighted section is the portion of the sequence matching the potato RNAi transgene. Upper case indicates translated sequence, lower case untranslated. Start sites are underscored in unaligned nt sequences; (**B**) Alignment of the RNAi transgene regions. Translation start sites are boxed. Lines between nucleotides indicate identity, highlighting indicates continuous regions of nucleotide identity. Translated sequence is not included with nucleotides and translation is shown without alignment because all translated residues are identical.

A single *C. maculata* sequence with very high similarity to the translated sequence encoding the lethal actin dsRNA *ACT* from *L. decemlineata* was found in each of the two transcriptomes. The two putative *C. maculata* sequences were identical in nucleotide sequence for 1505 nt. The translation of the two sequences identified a predicted full length protein of 376 aa (Figure 2A). The predicted *C. maculata* actin sequence (CmacAct) was aligned with the 297 nt used to develop transgenic insect resistant potato plants. The sequences and alignments of nt sequences are shown in Figure 2B. The aa identity was 100% and the nt identity was 257/296 or 87%. The nt alignment resulted in four continuous nt identities of 17 nt or longer, and these longer regions were adjacent to other identical regions (separated by one non-identical nt) (Figure 2B). There were no identical regions longer than 20 nt.

Eleven sequences from the *T. castaneum* genome predicted as prime target genes for RNAi disruption [13] were used to seek similar sequences in the *C. maculata* transcriptomes. The search results are summarized in Table 1; three of the sequences did not result in a probable match, but the other eight were highly similar to both the aa and nt sequences from unique predicted genes. Each pair of predicted *C. maculata* sequence encoded at least one full length translation; one sequence predicted four isoforms (the contigs similar to L82/*gw*, predicted reference sequence XM_015982857). Only one of the eight predicted gene sequences, the one similar to L55/*pp1 alpha-96a*, predicted reference sequence XM_001813922, did not contain a region of 17 or more contiguous nucleotide identities.

## 4. Discussion

RNAi was initially described as an intracellular, or cell autonomous, process. It was soon further elucidated as a systemic process within multicellular organisms, and then shown to function through external exposure via feeding or soaking an organism [12]. Introduction of dsRNA to an organism from an external source has been termed “environmental RNAi” (eRNAi) [19]. The potential for eRNAi as a crop protection strategy was recognized and tested in nematodes [20,21] and insects [2,22] soon after demonstration in model organisms. Progression to a practical field implementation against the target insect *D. v. virgifera* followed rapidly [3,4,6], and research progressed to include addressing concerns of impact to non-target organisms. A thorough set of studies to assess risk to non-target arthropods concluded in field tests that the presence of beneficials, identified as predatory earwigs, lacewings, lady beetles, minute pirate bugs, parasitic wasps, and spiders, in statistically similar quantities indicated no risk [23]. Plot sizes designated for the counts were less than 100 m^2^, and the life stage of arthropods counted was not specified. Studies have also included laboratory bioassays, and *C. maculata* was one of the laboratory test species for risk assessment. When treated with diet incorporating the *Snf7* dsRNA, results conclusively demonstrated no ill effects [3]. For lady beetles, there is wide variation among species in dietary habits. Most are predatory, although the Mexican bean beetle, *Epilachna varivestis* Mulsant (Coleoptera: Coccinellidae), is a serious pest and feeds on living plant tissue. While some predatory lady beetles specialize on a single prey species, some are more catholic in dietary choices. The North American native species (or species complex) *C. maculata* is known to consume pollen as a substantial portion of its diet [10,24]. This species, a logical choice for non-target testing in North America, was included as one of the non-target arthropods for environmental risk testing. The sequence comparison provided here and shown in Figure 1B provides further demonstration that the dsRNA used for transgenic corn rootworm resistant maize varieties should not interfere with the predicted CmacSnf7 gene. The nt alignment did not result in any continuous nt identities of >17 nt, a predicted minimal length suggested for predicting off-target effects [13,18]. In fact, the longest series of nt identities of the sequences was 9. While predicting off-target effects by searching for 17+ continuous nt similarity is not a certainty, particularly when considering diverse organisms such as insects, short RNAs as small as 17 nt may produce gene interference in some insects [13].

In a series of innovative experiments, dsRNA constructs were inserted into the genome of potato plant chloroplasts [7]. This strategy increased the likelihood of delivery of the effective long dsRNAs to the target pest, *L. decemlineata*, and was shown to be highly lethal [7]. A dsRNA similar to the *D. v. virgifera* sequence *Snf7*, *SHR*, was less lethal than a dsRNA from a portion of the sequence from an actin gene [7]. The CmacSnf7 sequence had even less identity to the 220 nt sequence tested in *L. decemlineata*, *SHR*, 142/212 or 67% (compare to Figure 1B). The longest identical nt segment was 12 nt (not shown). However, the actin gene used in the experiments was very similar to the CmacAct gene identified from transcriptomes. While there were no identical regions longer than 21 nt, the canonical effective siRNA length, the region between nt 60 and nt 115 has only four mismatches. This similarity warrants further risk assessment studies if the actin gene sequence is further implemented as a crop protection strategy. *C. maculata* is known to consume the eggs of *L. decemlineata*, and in a potato agroecosystem it is possible that the pest eggs could constitute substantial portion of the beneficial lady beetles’ diet. While it could be argued that eggs have not fed and could not contain plant-derived dsRNA, it could also be possible that a female adult *L. decemlineata* feeding on dsRNA could transfer some portion of the dsRNA to eggs during oogenesis, exposing the beneficial insect. More disturbingly, the *L. decemlineata ACT* sequence is closely identical to a sequence on file in GenBank for a species of commercially produced beneficial generalist parasitoid, *Trichogramma pretiosum* Riley (Hymenoptera: Trichogrammidae), with continuous nt identity regions up to 32 nt long (sequence XM_014379004.1).

For future RNAi pest control development, a robust screen of the genetic model insect *T. castaneum* indicated some categories of genes and eleven specific candidate genes for use [13]. Direct comparison of the eleven candidate genes clearly identified eight predicted orthologs in *C. maculata* (Table 1). While some of the identified transcriptome sequences were not precisely alike in the two specimen assemblies, the variations could be explained by minor sequencing error, different alleles containing nucleotide polymorphisms, or diet-induced expression variation. The three genes that were not found could be present in the *C. maculata* genome but were not expressed in the adult stage that was used for transcriptome sequencing. The 17+ nt identity occurrences between two distantly related beetles, *T. castaneum* and *C. maculata*, suggests that careful analysis of non-target species when choosing target genes is warranted. That being said, the eight predicted potential target genes compared in Table 1 were long enough to provide ample portions of sequence for dsRNA construction while avoiding those sites with non-target nt identity. Sequencing of the genomes or transcriptomes of an increasing number of non-target species should be undertaken to support good decision making. Decisions concerning non-targets for sequencing or bioassays could be assisted by tools such as the database described for use in portions of Europe [25]. An elegant tool designed to compare RNAi targets for one species against other species was developed [26]. However, without available sequences from beneficial and benign species the program has limited utility.

## 5. Conclusions

The analyses performed here, sequence comparisons based on transcriptome data from a limited, highly inbred sample, can neither guarantee biosafety nor prove environmental risk. Nonetheless, the close identity of actin sequences demonstrated by this analysis may serve as an illustration for selection of target genes intended for commercialization. There is an enormous quantity of potential RNAi targets for use in both research and agricultural implementation. The selectivity and specificity of RNAi has great potential, as future research and development will doubtless prove. Further sequencing of non-target organisms will speed up and enhance target gene choice and support risk assessments.

## Figures and Tables

**Table 1:** Eleven novel RNAi target genes from *Tribolium castaneum* compared with *Coleomegilla maculata* predicted homologue sequences.

Symbol/Name	Refseq ID	Sequence Identification from *C. maculata* Transcriptomes			*T. castaneum* vs. *C. maculata*		
		Pollen-Fed	Insect Egg-Fed	Similarity (*C. mac*)	aa Identities	nt Similarity	17+ nt Matches
L10/*cact*	NM_001163711	not found	not found	n/a	n/a	n/a	n/a
L11/*srp54k*	XM_962796	comp3017	comp3093	1558/1558	474/508	1104/1415 (78%)	2
L44/*rop*	NM_001170684	comp12493	comp11584	2442/2449	528/590	1321/1781 (74%)	4
L47/*alpha snap*	XM_968056	comp8167	comp5787	2338/2338	212/241	651/879 (74%)	1
L50/*shi*	XM_008200600	not found	not found	n/a	n/a	n/a	n/a
L55/*pp1alpha-96a*	XM_001813922	comp10238	comp10443	1751/1752	318/327	781/986 (79%)	0
L67/*inr-a*	XM_008194324	not found	not found	n/a	n/a	n/a	n/a
L76/*hsc70-3*	XM_008202764	comp14394	comp13114	2433/2488	598/645	1528/1924 (79%)	8/7
L80/*rpn7*	XM_968550	comp12477	comp14743	1296/1301	322/389	849/1173 (72%)	1
L82/*gw*	XM_015982857	comp8599	comp10202	4423/4424	967/1388	1781/2609 (68%)	11
L84/*rpt3*	XM_962883	comp3229	comp12416	1406/1434	393/409	907/1211 (75%)	1
